# Commentary: Permanent pacemaker implantation after cardiac surgery: Are we getting distracted by the usual suspects?

**DOI:** 10.1016/j.xjon.2021.06.025

**Published:** 2021-07-02

**Authors:** Filippo Rapetto, Vito D. Bruno

**Affiliations:** Division of Cardiac Surgery, Bristol Heart Institute, Bristol, United Kingdom; Translational Health Sciences, University of Bristol, Bristol, United Kingdom

**Figure:**
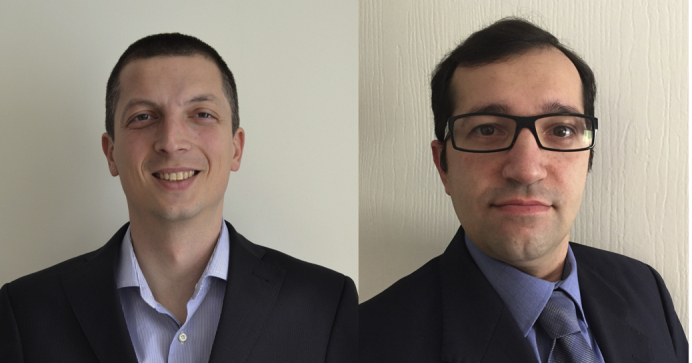
Filippo Rapetto, MD, and Vito D. Bruno, MD, PhD

**Central Message**

Evaluating the influence of pacemaker implantation after cardiac surgery is challenging. Many important confounders and different times of exposure might have relevant influence on the outcomes.

See Article page 157.

Arrhythmias requiring permanent pacemaker (PPM) implantation are recognized complications after cardiac surgery. Bianco and colleagues^1^ report their analysis of the influence of postoperative PPM implantation on clinical outcomes following isolated and combined valve surgery at their institution.

The authors' conclusion that PPM implantation is not an independent predictor of worse outcomes seems logical and counterintuitive at the same time. On the one hand, the article clearly shows that patients who need a PPM postoperatively are a higher risk group than their counterpart; moreover, various multivariable models unmask the fact that PPM implantation is probably a surrogate marker of the burden of comorbidities, which are the ultimate determinants of the patients' outcome. On the other hand, PPM implantation is a known risk factor for the development of serious complications, such as endocarditis, tricuspid regurgitation, and pacemaker-induced cardiomyopathy.^2^, ^3^, ^4^ Clearly, each of these adverse events can affect hospital readmission rate, and also late survival. How is this possible? PPM-related complications cannot have simply disappeared in the study by Bianco and colleagues.^1^ Hence, some other clinical factors might have played a role.

To explain this apparent discrepancy, 3 factors must be considered: age, time, and the usual suspects. The median age of the patients included in this study is 74 years and 70 years in the PPM and non-PPM groups, respectively, and the median follow-up time is 4.8 years. The study cohort is representative of the average patient undergoing cardiac surgery: in older patients, comorbidities (the usual suspects such as left ventricular dysfunction, chronic kidney disease, chronic obstructive pulmonary disease, diabetes mellitus, and immunosuppression) play a bigger role than the relatively uncommon PPM-related complications. Furthermore, it is possible that the relatively short follow-up did not allow for a significant number of PPM-related complications to develop, whereas preexisting comorbidities can even be exacerbated by cardiac surgery itself. This is indirectly confirmed by previous articles with similar design, similar study population, longer follow-up, and opposite conclusions.^5^^,^^6^

In other words, what would happen if we followed-up younger patients, for a longer period of time and with less interference from the usual suspects? Probably, we would find that PPM-related complications have a significant influence. Similarly, it is widely recognized that valve prosthesis-related complications (including mortality) are extremely more significant in young patients, whose long survival implies an increase in the cumulative incidence of adverse events.^7^^,^^8^

As always in medicine, the central question is not “Is this treatment good?” but rather, “Is this treatment good for this patient?” The message from Bianco and colleagues^1^ is very important because it applies to the most common patients that cardiac surgeons operate on in their daily practice, and at least for the first few years after surgery. However, it should not lead us to underestimate the potential implications of postoperative PPM implantation in different—but equally important—patient populations in which improving the long-term outcomes is of paramount importance.
